# Emerging *Bordetella pertussis* Strains Induce Enhanced Signaling of Human Pattern Recognition Receptors TLR2, NOD2 and Secretion of IL-10 by Dendritic Cells

**DOI:** 10.1371/journal.pone.0170027

**Published:** 2017-01-11

**Authors:** Elise S. Hovingh, Marjolein van Gent, Hendrik-Jan Hamstra, Marc Demkes, Frits R. Mooi, Elena Pinelli

**Affiliations:** Centre for Infectious Disease Control, National Institute for Public Health and the Environment, Bilthoven, The Netherlands; Universidad Nacional de la Plata, ARGENTINA

## Abstract

Vaccines against pertussis have been available for more than 60 years. Nonetheless, this highly contagious disease is reemerging even in countries with high vaccination coverage. Genetic changes of *Bordetella pertussis* over time have been suggested to contribute to the resurgence of pertussis, as these changes may favor escape from vaccine-induced immunity. Nonetheless, studies on the effects of these bacterial changes on the immune response are limited. Here, we characterize innate immune recognition and activation by a collection of genetically diverse *B*. *pertussis* strains isolated from Dutch pertussis patients before and after the introduction of the pertussis vaccines. For this purpose, we used HEK-Blue cells transfected with human pattern recognition receptors TLR2, TLR4, NOD2 and NOD1 as a high throughput system for screening innate immune recognition of more than 90 bacterial strains. Physiologically relevant human monocyte derived dendritic cells (moDC), purified from peripheral blood of healthy donors were also used. Findings indicate that, in addition to inducing TLR2 and TLR4 signaling, all *B*. *pertussis* strains activate the NOD-like receptor NOD2 but not NOD1. Furthermore, we observed a significant increase in TLR2 and NOD2, but not TLR4, activation by strains circulating after the introduction of pertussis vaccines. When using moDC, we observed that the recently circulating strains induced increased activation of these cells with a dominant IL-10 production. In addition, we observed an increased expression of surface markers including the regulatory molecule PD-L1. Expression of PD-L1 was decreased upon blocking TLR2. These *in vitro* findings suggest that emerging *B*. *pertussis* strains have evolved to dampen the vaccine-induced inflammatory response, which would benefit survival and transmission of this pathogen. Understanding how this disease has resurged in a highly vaccinated population is crucial for the design of improved vaccines against pertussis.

## Introduction

*Bordetella pertussis* is a human-specific Gram-negative bacterium and the causative agent of whooping cough, also known as pertussis. This highly contagious disease of the upper respiratory tract can be fatal in unvaccinated infants and is the cause of persistent cough among older children and adults [1]. Whole cell pertussis vaccines (WCVs) were introduced worldwide in the 1950’s after which the number of notifications decreased dramatically [2]. WCVs were relatively reactogenic and therefore replaced by acellular pertussis vaccines (ACVs) in the 1990’s in most Western countries [3–5]. In the Netherlands, WCVs were introduced as part of the national immunization program in 1957 and replaced by ACVs in 2005 [6]. Despite high vaccination coverage, cases of pertussis have increased in the past three decades [7]. Possible explanations for this resurgence include improved diagnosis, increased awareness, waning of vaccine-induced immunity and genetic changes of *B*. *pertussis* (reviewed in [8]). Observed bacterial changes include the emergence of strains that carry novel alleles for *prn*, *fim2*, *fim3*, *ptxA* and *ptxP* as well as the non-expression of the vaccine antigen pertactin (Prn) [9–11]. A mix of *ptxP1* (P1) and *ptxP2* (P2) strains were prevalent before the introduction of the WCVs and were used to produce the pertussis vaccines. After the introduction of the WCVs mainly P1 strains circulated [12, 13]. The *ptxP3 (*P3) strains emerged in the 1980’s and represent more than 90% of the current *B*. *pertussis* populations in many countries worldwide [9, 14]. These P3 strains have been associated with the resurgence and severity of pertussis [11, 15, 16].

Dendritic cells (DCs) are innate immune cells that sense pathogens by means of pattern recognition receptors (PRRs) including the Toll-like receptors (TLRs) and the NOD-like receptors [17–19]. Each receptor recognizes a unique pathogenic molecular structure resulting in a cascade of events leading to NF-κB activation and subsequent cytokine production [20]. Activation of different PRRs by pathogens is essential for triggering innate immunity and for the induction and steering of the subsequent adaptive immune response [21, 22].

Although *B*. *pertussis* strain adaptation has been extensively studied [23], little is known about the effect that these bacterial changes may have on innate immune responses to this pathogen. Here, we characterize PRR and moDC activation induced by genetically diverse *B*. *pertussis* clinical isolates circulating before and after the introduction of the pertussis vaccines. We observed that all *B*. *pertussis* clinical isolates induce TLR2, TLR4 and NOD2 but not NOD1 signaling in HEK-Blue cells. In addition, we observed an increased activation of TLR2 and NOD2 by strains circulating after, compared to before, the introduction of the pertussis vaccines. Furthermore, when using moDCs, we observed that recently circulating strains, particularly the ones that emerged after the introduction of ACVs, induced stronger DC activation resulting in a dominant increased production of the anti-inflammatory cytokine IL-10 and increased expression of surface markers including the regulatory molecule PD-L1.

These *in vitro* studies suggest that emerging *B*. *pertussis* strains have evolved to induce a more regulatory response, which could be an immune evasion strategy used by this pathogen to increase its survival and transmission in a highly vaccinated population.

## Materials and Methods

### Ethics approval

This study was conducted using blood donations for primary cell isolation, a research goal not requiring review by an accredited Medical Research Ethics Committee (MREC), as determined by the Dutch Central Committee on Research Involving Human Subjects (CCMO) (http://www.ccmo.nl/en/help-me-on-my-way-static) and warranted by a solicited accredited MREC. The study was conducted according to the principles expressed in the Declaration of Helsinki and written informed consent was obtained from all blood donors before collection and anonymous use of their samples.

### *Bordetella pertussis* clinical isolates

**Fig 1:**
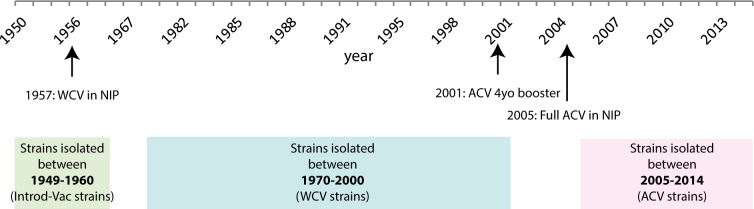
Schematic representation of the time periods in which the strains used in this study were grouped, based on their year of isolation. The periods included strains isolated before or around the time the WCVs were introduced (Introd-Vac-strains, 1949–1960) and after the introduction of the WCVs (WCV-strains, 1970–2000) or ACVs (ACV-strains, 2005–2014). The three groups are indicated in green, blue and pink respectively. The WCV was introduced in the Dutch National Immunisation programme (NIP) in 1957. In 2001, the ACVs were included in the NIP as a four year old (4yo) booster. The WCVs were fully replaced by the ACVs in 2005 (black arrows).

In this study, ninety-two *B*. *pertussis* strains isolated from pertussis patients were randomly selected from the *B*. *pertussis* strain collection at the National Institute for Public Health and the Environment, The Netherlands. As shown in Fig 1, the strains were divided into three groups according to year of isolation: 1) from 1949 to 1960; a period just before and during the time WCVs were introduced (Introd-Vac-strains), 2) from 1970 to 2000; a period after the introduction of WCVs (WCV-strains) and 3) from 2005 to 2014; after the introduction of ACVs (ACV-strains). Table 1 gives an overview of all the strains used in this study, their year of isolation and the *ptxP*, *prn*, *fim2*, *fim3* and *ptxA* allele type, which was previously determined [24]. These strains were incubated with the different HEK-Blue cell lines expressing either TLR2, TLR4, NOD1 or NOD2. In addition to grouping the strains according to their year of isolation, the strains were also grouped based on the *ptxP* allele they carried (P1, P2 and P3). For the experiments using the moDCs, 29 strains were selected from the 92 strains, as representative for each of the periods mentioned above. The selection was made based on the PRR activation levels that the selected strains induced, compared to the PRR activation levels of all 92 strains. As shown in S1 Table, the average SEAP-OD value from the selected strains did not deviate more than 15% from the average OD value of all tested strains per group and PRR. From these 29 strains, six Introd-Vac-strains and six ACV-strains were used to determine their effect on DC maturation as indicated by expression of activations markers on the moDC cell surface. Six additional Prn-deficient strains were included in the moDC experiments. The *B*. *pertussis* laboratory strain Tohama I (B0213; streptomycin and naladixic acid resistant) was also included in this study to normalize for possible plate differences. All *B*. *pertussis* isolates were grown at 35°C on Bordet Gengou (BG) plates containing glycerol and 15% defibrinated sheep blood (BD Biosciences). After three days of culture, the bacteria were collected in phosphate-buffered saline (PBS, Gibco), and the optical density (OD) was measured at 600 nm.

**Table 1:** *B*. *pertussis* strains used in this study isolated from Dutch patients between 1949 and 2014.

Strain	Year of Isolation	*ptxP*	*Prn_region1*	*Prn_region2*	*fim2*	*fim3*	*ptxA*	Group
B0558	1949	1	1		1	1	2	1950–60
B1195	1949	1	1		1	1	2	1950–60
B0304	1950	1	1				1	1950–60
B0339	1950	1				1	2	1950–60
B0560	1950	2	1	3		1	4	1950–60
B0562	1950	1	2			1	1	1950–60
B0565	1950	1	1			1	2	1950–60
B1197	1950	1	1			1	2	1950–60
B1198	1950	2	7		2	1	4	1950–60
B1200	1950	1	1			1	2	1950–60
B0335	1951	2	1	3	2	1	4	1950–60
B0338	1951	2		3			4	1950–60
B0566	1951	2	1	3			4	1950–60
B0567	1951	2	7		2	1	4	1950–60
B0569	1951	2	1	3			4	1950–60
B0296	1952		1			1	2	1950–60
B0336	1952	1	1		1	1	2	1950–60
B0441	1952	2	7		2	1	4	1950–60
B0571	1952	1	1			1	2	1950–60
B1203	1952	1	1			1	2	1950–60
B0443	1953	1	1			1	2	1950–60
B1363	1955	2	7		2	1	4	1950–60
B1377	1955	2	1	3		1	4	1950–60
B1378	1955	2	1	3	2	1	4	1950–60
B1381	1956	1	1			1	2	1950–60
B1382	1957	2	1	3		1	4	1950–60
B1383	1957	1	1		1	1	2	1950–60
B1384	1957	2	1	3		1	4	1950–60
B1365	1958	1	1		1	1	2	1950–60
B0440	1978	1	1		1	1	1	1970–2000
B0678	1981	1	1			1	1	1970–2000
B2939	1985	1	1	1		1	1	1970–2000
B0760	1987	1	1			1	1	1970–2000
B2965	1987	1	1			1	1	1970–2000
B2969	1987	1	1			1	1	1970–2000
B0834	1988	1	1			1	1	1970–2000
B0861	1989	1	3				1	1970–2000
B0870	1990	1	2			1	1	1970–2000
B0556	1993	1	2			1		1970–2000
B0346	1994	1	3			1		1970–2000
B0354	1994	1	3			1		1970–2000
B0520	1994	1	3			1	1	1970–2000
B0538	1994	1	3					1970–2000
B0541	1994	3	2				1	1970–2000
B0597	1995	1	3			1	1	1970–2000
B0599	1995	1	3			1	1	1970–2000
B0604	1995	1	3			1	1	1970–2000
B0606	1995	3	2			1	1	1970–2000
B0611	1995	1	3				1	1970–2000
B0661	1996	1	3					1970–2000
B1722	1997	1	1			1	1	1970–2000
B1781	1997	3	2	1		1	1	1970–2000
B1707	1998	3	2				1	1970–2000
B1805	1998	3	2	1		1	1	1970–2000
B1960	1998	3	2					1970–2000
B1826	1999	1	2			1	1	1970–2000
B1895	2000	3	2					1970–2000
B1917	2000	3	2	1	1	2	1	1970–2000
B1927	2000	3	2					1970–2000
B2911	2005	3	2		1	1	1	2005–14
B2914	2005	3	2			1	1	2005–14
B3104	2007	3	2		1	1	1	2005–14
B3107	2007	3	2	1	1	1	1	2005–14
B3115	2007	3	2	S	1	1	1	2005–14
B3130	2007	3	2	1	1	1	1	2005–14
B3134	2007	3	2		1	2	1	2005–14
B3135	2007	3	2	1	1	1	1	2005–14
B3173	2007	3	2	1	1	1	1	2005–14
B3180	2008	3	2		1	1	1	2005–14
B3203	2008	3	2		1	2	1	2005–14
B3212	2008	1	2		1	1	1	2005–14
B3235	2008	13	2	1	1	1	1	2005–14
B3269	2008	3	2	1	1	1	1	2005–14
B3274	2008	3	2	1	1	1	1	2005–14
B3339	2009	3	2		1	1	1	2005–14
B3392	2009							2005–14
B3400	2009							2005–14
B3446	2010	3	2	1	1	1	1	2005–14
B3894	2011	3	2	1	1	1	1	2005–14
B3896	2011	3		1	1			2005–14
B3903	2011	3	2	1		1		2005–14
B3909	2011	3	2	1	1	1		2005–14
B3912	2012	3	2	1	1	1		2005–14
B3915	2012	3	2	1	1	1	1	2005–14
B3916	2012	3	2	1	1	1	1	2005–14
B3924	2012	1	1	1	1			2005–14
B3940	2012							2005–14
B3951	2012	3	2	1	1	1	1	2005–14
B3962	2012	3	2	1	1	1	1	2005–14
B3967	2012							2005–14
B4011	2012	3	2	1				2005–14
B4171	2014	3	2	1	1	1	1	2005–14
B3640	2010	3	2	1	1	1	1	Prn-
B3645	2007	3	2	1	1	1	1	Prn-
B3865	2011	3	2		1	1	1	Prn-
B3876	2011	3	2	1	1	1	1	Prn-
B3977	2012	3	2	1	1	1	1	Prn-
B4172	2014	3	2	1	1	1	1	Prn-

Table 1: Strains were divided into three groups according to year of isolation: strains isolated before and around the time of the WCVs were introduced in the Netherlands (1950–1960, Introd-Vac-strains), strains isolated during the time the WCVs were used (1970–2000, WCV-strains) and strains isolated during the time the ACVs were used (2005–2014, ACV-strains). Alleles for the *ptxP*, *prn* region one and two, *fim2*, *fim3* and *ptxA* determined by sequencing are indicated in the table, when known.

### HEK-Blue cell lines and stimulation

To determine which human PRRs are activated by the different *B*. *pertussis* clinical isolates, live bacteria were incubated at a multiplicity of infection (MOI) of 40 with the NF-κB/SEAP reporter HEK293-Blue (HEK-Blue) cell lines expressing either TLR2 (HEK-Blue-TLR2), TLR4/MD-2/CD14 (HEK-Blue-TLR4), NOD-1 (HEK-Blue-NOD-1) or NOD-2 (HEK-Blue-NOD2) (InvivoGen, San Diego, California,USA). To control for endogenous PRR activation, un-transfected parental HEK-Blue-Null1 cells were also used. All cell lines contained an NF-kB-inducible secreted embryonic alkaline phosphatase (SEAP) reporter gene. PRR signaling leads to the expression of SEAP, which can be detected in culture supernatants upon addition of the substrate Quanti-Blue (InvivoGen). The HEK-Blue cells were cultured as previously described [25]. In short, HEK-Blue cells were stimulated in a volume of 200 μl in 96-wells plates for 24 hours according to the manufacturer’s instructions. An MOI of 40 was used as the optimal bacterial concentration based on previous titration experiments using 10-fold bacterial dilutions. With a MOI of 40 all HEK-PRR cells responded with SEAP activity values that fell within the linear range response obtained using the respective positive controls. The experiment was performed on three separate days and to correct for possible plate and day differences, the laboratory strain Tohama I was taken along on each plate. PRR activation by the different *B*. *pertussis* clinical isolates was expressed as the SEAP activity induced normalized to the activity induced by the *B*. *pertussis* laboratory strain Tohama I.

### Purification, culture and stimulation of moDCs

MoDcs were purified from peripheral blood derived from healthy donors and cultured as described previously [25]. Briefly, after peripheral blood mononuclear cell isolation using a Lymphoprep (Nycomed, Zurich, Switzerland) gradient, monocytes were collected by using MACS in combination with anti-CD14 microbeads (Miltenyi Biotech, Bergisch Gladbach, Germany). Purity of the CD14-positive cells was determined by staining the monocytes with anti-CD14-PE (BD biosciences,) followed by data acquisition on the FACS Canto II (BD Biosciences) and analysis using FlowJo software (Tree Star). CD14 positive purity was more than 90% for each donor. Monocytes were cultured in 24-well culture plates at 0.4 x 10^6^ cells/well for 6 days in IMDM culture medium supplemented with 1% FCS, 100 units penicillin, 100 units streptomycin, and 2.92 mg/ml L-glutamine (Gibco; DC culture medium), and in the presence of 500 U/mL GM-CSF (PreproTech) and 800 U/mL IL-4 (Active biosciences). On day 6, the immature moDCs were either left unstimulated in DC culture medium supplemented with 500 U/ml GM-CSF or stimulated with live *B*. *pertussis* at an MOI of 10 (suboptimal bacterial concentration based on previous titration experiments) in the presence or absence of 1 μg/ml polyclonal anti-TLR2 (Invivogen) that was added two hours before adding the *B*. *pertussis* strains. The experiment was performed using moDCs from three separate donors and the laboratory strain Tohama I was taken along to correct for possible donor differences. After 48 hours of incubation, supernatants were collected and filtered using Ultrafree-MC GV centrifugal filers with a pore size of 0.22 μm (Millipore), and stored at -80°C for cytokine analysis.

### FACS analysis

MoDCs stimulated with six Introd-Vac-strains or six ACV-strains were used to analyze surface marker expression. These moDC were stained with PE-conjugated anti-CD101 (BD Biosciences), APC-conjugated anti-CD97 (R&D systems), FITC-conjugated anti-CD80 (BD Biosciences), Pacific Blue-conjugated anti-CD86 (BioLegend), PerCP-eFluor710- conjugated anti-CD274, PE/Cy7-conjugated anti-CD172a/b and with LIVE/DEAD Fixable Aqua Dead Cell Stain Kit (Invitrogen) for 30 minutes at 4°C. The moDC were washed twice with FACS buffer (PBS pH 7.2; 0,5% BSA; 0,5 mM EDTA) and fixed with 1.5% PFA. Data was acquired on FACS Canto II (BD Biosciences) and analyzed using FlowJo software (Tree Star). More than 90% of the stimulated cells remained viable after the 48-hour stimulation.

### Cytokine analysis

The concentration of the cytokines IL-1β, IL-6, IL-10, MCP-1, MIP-1α, MIP-1β, and TNF-α was measured in supernatants from moDC that were stimulated for 48 hours, by using a Bio-rad human 8-plex luminex kit (Bio-Rad, Hercules, CA, USA) according to the manufacturer’s instructions. Measurements and data analysis were performed with Bio-Plex 200, using Bio-Plex Manager software (version 5.0; Bio-Rad Laboratories). IL-8 concentration was measured using an IL-8 ELISA kit (R&D, Minneapolis, MN, USA) which was performed according to the manufacturer’s instructions. Results are expressed as pg/ml of cytokine production induced by the different *B*. *pertussis* isolates normalized to the cytokine levels induced by the *B*. *pertussis* laboratory strain Tohama I, to correct for possible plate/donor differences.

### Statistical analysis

All statistical analyses were performed using GraphPad Prism 6.04. Statistical significance was determined by using ANOVA followed by unpaired t-tests. False discovery rate was controlled at the level of 10% by applying the Benjamini-Hochberg method to the results of all the tests performed. Results presented as statistically significant have passed the selection based on the False discovery rate. *P* values ≤0.05 were considered statistically significant.

## Results

### *Bordetella pertussis* strains isolated from patients after the introduction of pertussis vaccination induce increased activation of human TLR2 and NOD2

**Fig 2:**
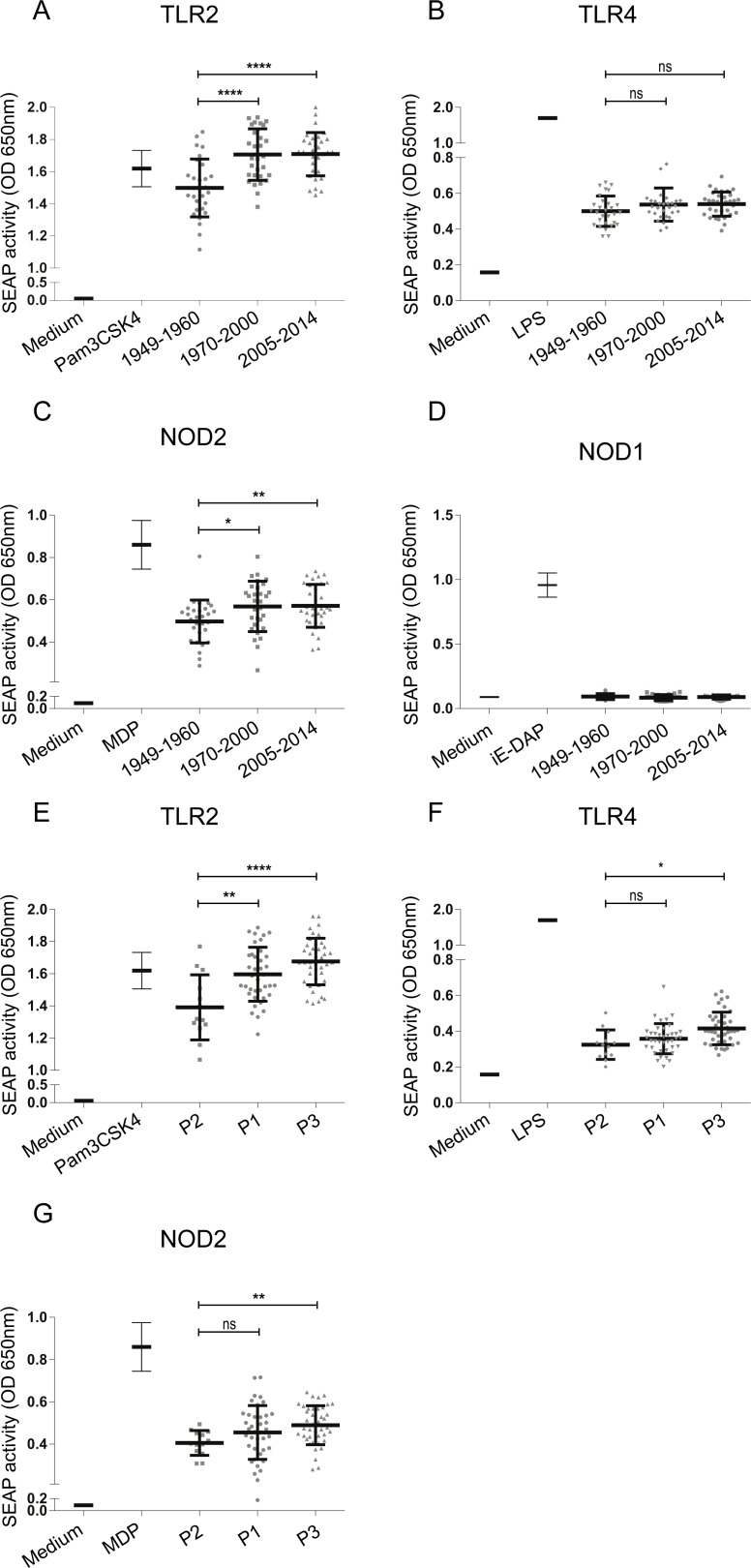
Activation of human TLR2, TLR4, NOD2 and NOD1 by *B*. *pertussis* strains isolated from patients from 1949 to 2014. HEK-Blue cell lines either expressing TLR2, TLR4, NOD2 or NOD1 were stimulated with 92 different *B*. *pertussis* strains (MOI 40) isolated from pertussis patients either around the time the WCVs were introduced (Introd-Vac-strains) or when the WCVs (WCV-strains) or ACVs (ACV-strains) were used. PRR activation measured by SEAP activity of (A) HEK-Blue-TLR2 cells, (B) HEK-Blue-TLR4 cells, (C) HEK-Blue-NOD2 cells or (D) HEK-Blue-NOD1 cells by Introd-Vac-strains (1949–1960), WCV-strains (1970–2000) and ACV-strains (2005–2014). SEAP activity induced by these same strains grouped according to their *ptxP* alleles, *ptxP1* (P1), *ptxP2* (P2) or *ptxP3* (P3), upon stimulation of (E) HEK-Blue-TLR2, (F) HEK-Blue-TLR4 cells or (G) HEK-Blue-NOD2 cells. SEAP activity of cells in medium only or with the respective ligands, Pam3CSK4 (100ng/ml), LPS-EK (10ng/ml), MDP (200ng/ml) or iE-DAP (100ng/ml) are included for each HEK-Blue cell line. * p ≤ 0.05, ** p ≤ 0.01, **** p ≤ 0.0001.

For this study, 92 *B*. *pertussis* strains isolated from pertussis patients were divided into three groups according to the year of isolation: 1) from 1949 to 1960; just before and during the time WCVs were first introduced, 2) from 1970 to 2000; after the introduction of WCVs and 3) from 2005 to 2014; after the introduction of ACVs in The Netherlands (Fig 1). Strains from these three periods were designated Introd-Vac-strains, WCV-strains and ACV-strains, respectively. In order to determine whether these strains induced TLR2, TLR4, NOD2 and NOD1 activation, HEK-Blue cells expressing either one of these PRRs were stimulated with the different *B*. *pertussis* clinical isolates. All of the tested strains activated TLR2, TLR4 and NOD2, but not NOD1 as indicated by the induced SEAP activity (Fig 2A–2D). Fig 2 shows that activation of TLR2 (Fig 2A) and NOD2 (Fig 2C) was significantly higher when stimulated with the WCV- as well as the ACV-strains compared to the Introd-Vac-strains. There was no significant difference in the activation of these PRRs between the WCV- and ACV-strains. No significant differences in TLR4 activation by the different group of strains were observed (Fig 2B). To control for non-specific activation all strains were incubated with the parental untransfected HEK-Null cells, which showed no induction of SEAP activity (S1 Fig). Upon grouping the strains according to the *ptxP* allele, into the groups P1, P2 and P3, we found significant increased TLR2 activation by P1 and P3 compared to the older P2 strains (Fig 2E). The P3 strains also showed an increased activation of NOD2 compared to P2 strains (Fig 2G) and a small but significant increase in TLR4 activation by P3 compared to the P2 strains was observed (Fig 2F). No difference between TLR2, TLR4 and NOD2 activation by P1 compared to P3 strains was detected. In order to determine whether mutations in *B*. *pertussis* gene sequences could explain the observed TLR2 activation differences, we compared the sequence of genes encoding the *B*. *pertussis* lipoproteins BP1569 and BP2992, which were recently shown to be TLR2 agonists [26]. From the strains used in this study, whole genome sequence data was only available for B1917, B0296, B0558 and B1281. Therefore, a Blast search of 340 *B*. *pertussis* whole genome sequences available in the NCBI database, which contains 60 Dutch strains, was performed [27]. Sequence data analysis did not reveal any polymorphism for the above mentioned *B*. *pertussis* lipoproteins genes in strains carrying the *ptxP1*, *ptxP2* or *ptxP3* alleles.

### Increased IL-10 production and regulatory surface marker expression by moDCs upon stimulation with *B*. *pertussis* strains isolated after the introduction of pertussis vaccines

**Fig 3:**
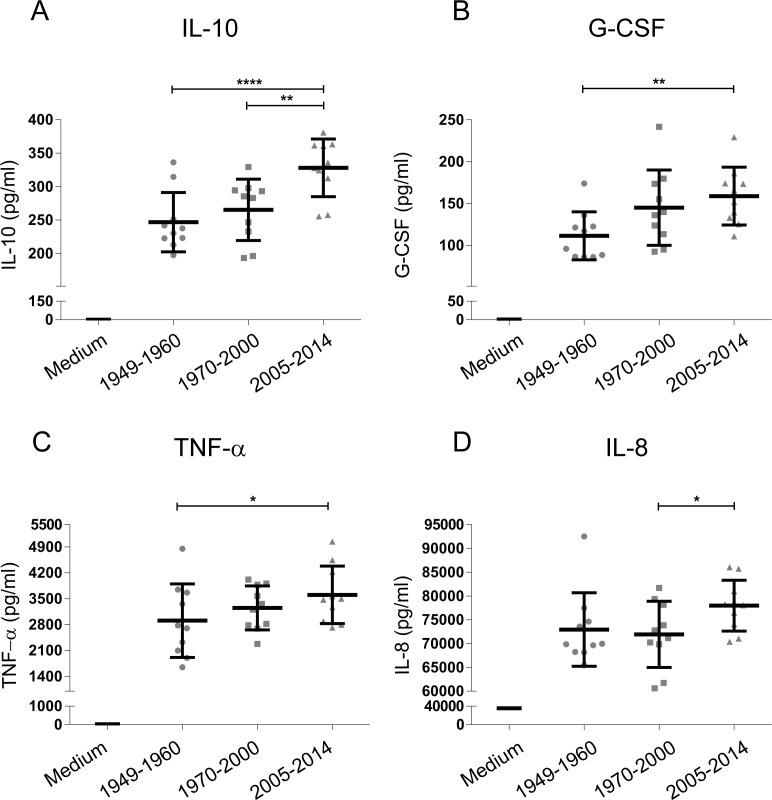
Cytokine production by moDCs stimulated with *B*. *pertussis* strains isolated from patients from 1949 to 2014. MoDCs were stimulated with 29 different strains either isolated around the time the WCVs were introduced (Introd-Vac-strains) or when the WCVs (WCV-strains) or ACVs (ACV-strains) were used at an MOI of 10. MoDC production of (A) IL-10, (B) G-CSF, (C) TNF-α and (D) IL-8 production by Introd-Vac-strains (1949–1960), WCV-strains (1970–2000) and ACV-strains (2005–2014). Medium stimulation values for IL-8, TNF-α, G-CSF and IL-10 are depicted in the appropriate graphs * p ≤ 0. 05, ** p ≤ 0.01, **** p ≤ 0.0001.

**Fig 4:**
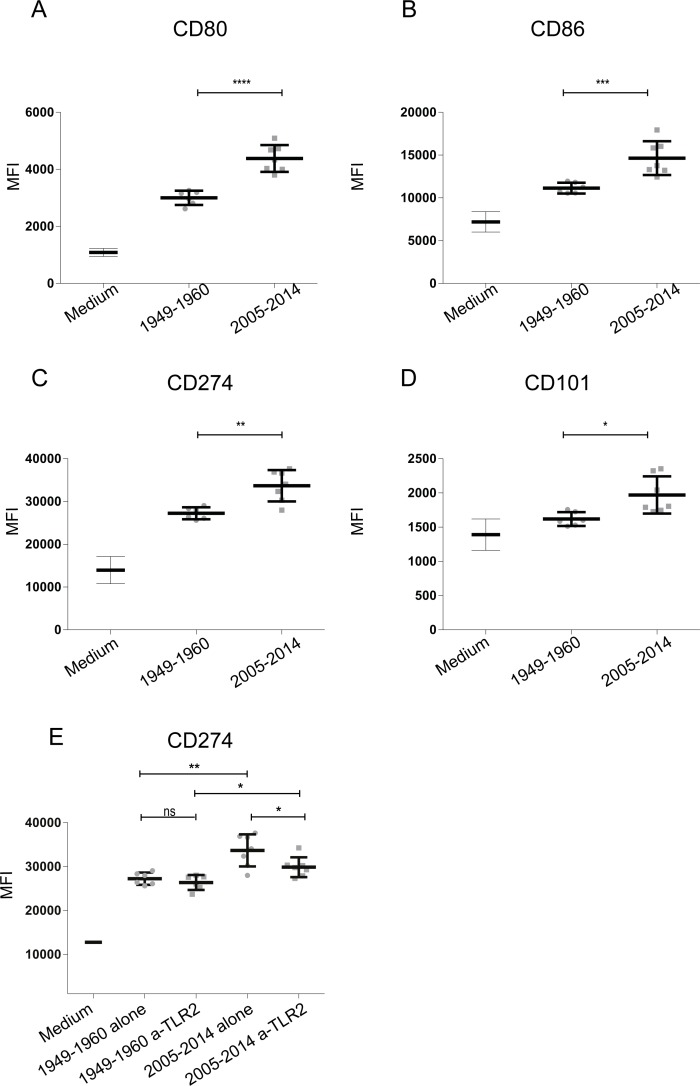
Surface marker expression of moDCs stimulated with Introd-Vac and ACV- *B*. *pertussis* strains isolated from pertussis patients. MoDCs were stimulated with 12 different *B*. *pertussis* strains (MOI 10) isolated from patients at around the time the WCVs were introduced (Introd-Vac-strains) or when the ACVs (ACV-strains) were used, either in the presence or absence of 1 μg/ml anti-TLR2 blocking antibody. MoDC surface expression of (A) CD80, (B) CD86, (C) PD-L1/CD274 and (D) CD101 by Introd-Vac-strains (1949–1960) and ACV-strains (2005–2014) alone. (E) MoDC surface expression of PD-L1/CD274 by Introd-Vac-strains (1949–1960) and ACV-strains (2005–2014) in the presence or absence of 1 μg/ml anti-TLR2 blocking antibody. Medium stimulation values for CD80, CD86, PD-L1/CD274 and CD101 are depicted in the corresponding graphs * p ≤ 0. 05, ** p ≤ 0.01, *** p ≤ 0.001, **** p ≤ 0.0001.

In addition to using the HEK-Blue reporter cells expressing individual PRRs to screen for innate immune activation by emerging *B*. *pertussis* strains, moDCs isolated from peripheral blood of healthy donors were used. These cells were stimulated with a selection of 29 *B*. *pertussis* strains representing the three different periods, based on the average PRR activation levels of the selected 29 strains compared to all strains. Results show increased moDC activation upon stimulation with the ACV- compared to Introd-Vac-strains, with a dominant production of IL-10 and to a lesser extent of G-CSF by these cells (Fig 3A and 3B). Moreover, ACV-strains induced a significant increased production of IL-10 compared to WCV-strains (Fig 3A). A small but significant increased production of TNF-α and IL-8 was also observed when comparing WCV-strains to Introd-Vac-strains and ACV-strains to WCV-strains respectively (Fig 3C and 3D). In addition to cytokine production, expression of the surface markers CD80, CD86, CD101, CD172a/b, CD274 (PD-L1) and CD97 on moDC stimulated with Introd-Vac-strains or ACV-strains was analyzed using flow cytometry. Fig 4A–4D shows a significant increase in CD80, CD86, PD-L1 and CD101 surface expression on moDC stimulated with ACV-strains compared to Introd-Vac-strains (Fig 4A–4D). As shown in Fig 4E, the expression of PD-L1 was decreased on moDCs stimulated with ACV-strains in the presence compared to the absence of the TLR2 blocker. No difference in the levels of the other surface markers or cytokines was observed in the presence of the blocking TLR2 antibody. The levels of other cytokines namely, IL-6, MCP-1, MIP-1α and MIP-1β, as well as the surface markers CD97 and CD172 showed no significance difference when moDCs were stimulated with the strains isolated from the different periods (S2 Fig). The levels of IL-1β and IL-12 were either very low or undetectable upon stimulation with the different *B*. *pertussis* strains.

### Recently circulating P3 strains induce increased IL-10 production compared to older P3 strains

**Fig 5:**
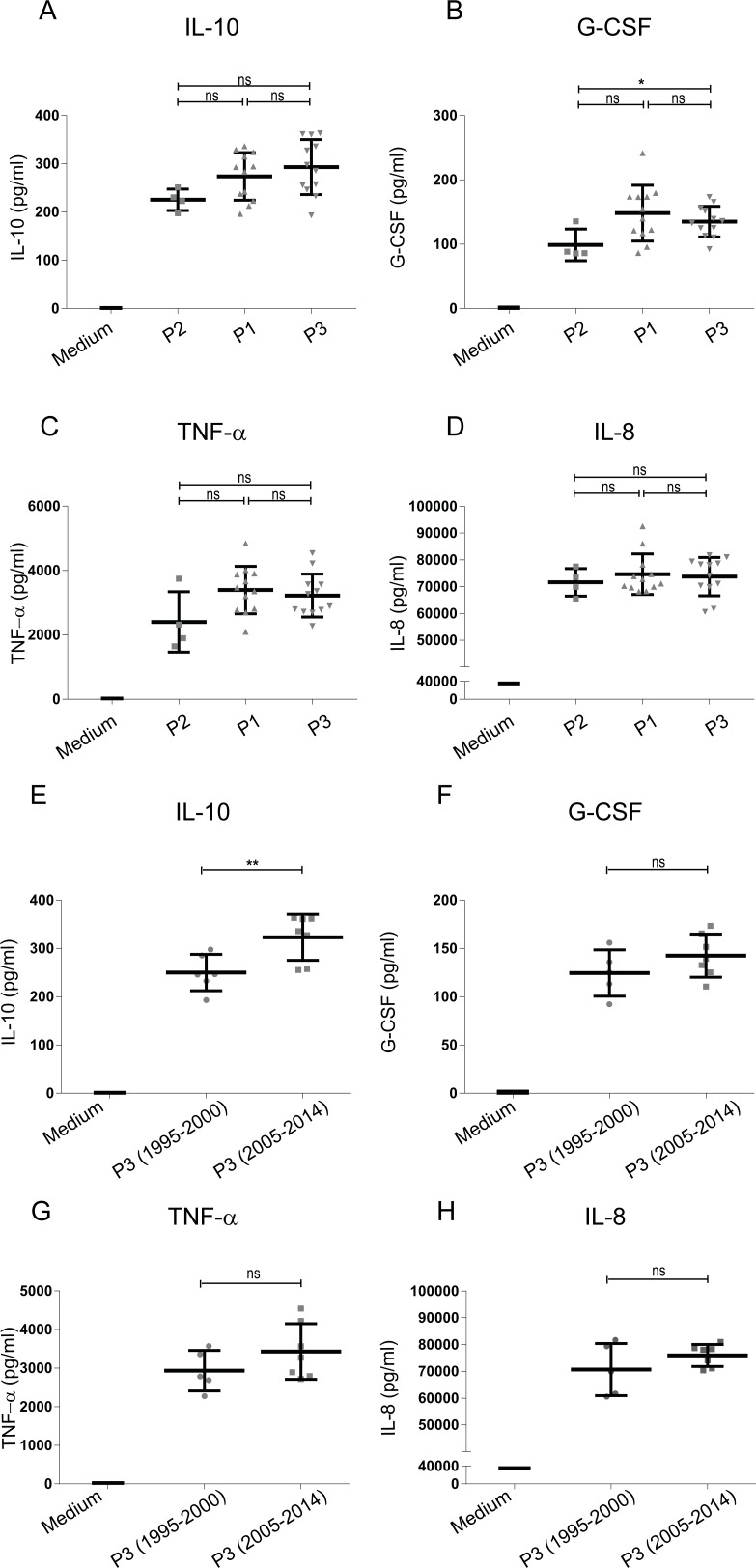
Cytokine production by moDCs stimulated with *B*. *pertussis* strains isolated from patients from 1949 to 2014 grouped according to *ptxP* allele. moDCs were stimulated with 29 different strains either expressing the *ptxP2* (P2), *ptxP1* (P1) or *ptxP3* (P3) allele, isolated around the time the WCVs were introduced (Introd-Vac-strains) or when the WCVs (WCV-strains) or ACVs (ACV-strains) were used at an MOI of 10. MoDC production of (A) IL-10, (B) G-CSF, (C) TNF-α and (D) IL-8 production by P2, P1 and P3 strains. The production of (E) IL-10, (F) G-CSF, (G) TNF-α and (H) IL-8 upon stimulation of MoDC by P3 strains isolated from 1995–2000 or P3 strains isolated from 2005–2014 was also investigated. Medium stimulation values for IL-8, TNF-α, G-CSF and IL-10 are depicted in the appropriate graphs * p ≤0. 05, ns = non-significant.

Characterization of genetically diverse groups of *B*. *pertussis* strains that have emerged over time have been previously reported [28]. Based on the sequence of the carried *ptxP* allele several groups of *B*. *pertussis* strains can be distinguished [29]. Older strains generally carry the P2 allele, whereas 90% of the currently circulating strains carry the P3 allele [9]. To determine whether *ptxP* allele carriage is associated with the observed moDC cytokine production induced by emerging *B*. *pertussis* strains, we grouped the strains according to their *ptxP* allele in the groups P2, P1 and P3. Results indicate that there were no significant differences in IL-10 (Fig 5A), TNF-α (Fig 5C) or IL-8 (Fig 5D) production by moDC when comparing the strains carrying different *ptxP* alleles. A significant increase in G-CSF production induced by P3 strains compared to P2 strains was observed (Fig 5B). Notably, when comparing the P3 strains isolated before (n = 5) and after the introduction of the ACV (n = 7), we observed that the more recently isolated P3 strains induced a significantly increased production of IL-10 by moDCs (Fig 5E) and an increased trend, although not significant, in activation of TLR2 and NOD2 (S3 Fig). No difference in the levels of G-CSF, TNF-α or IL-8 production was detected upon stimulating moDCs with the older vs recently circulating P3 strains (Fig 5F–5H).

### Prn-deficient *B*. *pertussis* strains induce a comparable moDC cytokine profile as Prn-expressing strains

**Fig 6:**
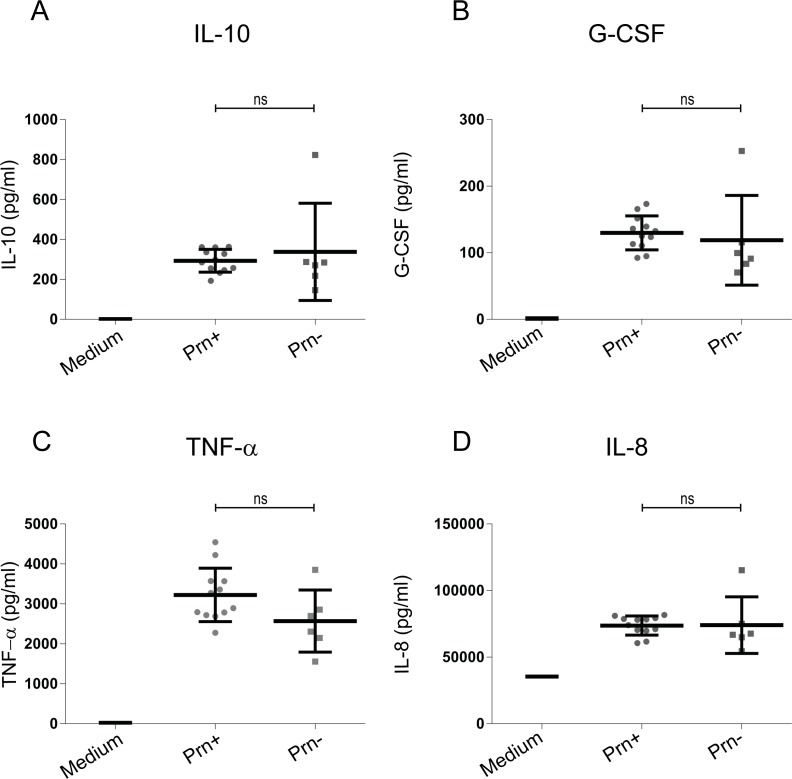
Cytokine production by moDCs stimulated with recently circulating Prn deficient or Prn expressing *B*. *pertussis* strains isolated from patients. MoDC production of (A) IL-10, (B) G-CSF, (C) TNF-α and (D) IL-8 production upon stimulation with P3 strains either expressing (Prn+) or not expressing Prn (Prn-). Medium stimulation values for IL-8, TNF-α, G-CSF and IL-10 are depicted in the corresponding graphs. ns = non-significant.

In recent years, *B*. *pertussis* P3 strains that do not express the vaccine antigen Prn have been emerging in countries that use the ACVs [30–34]. In some countries, more than 80% of the strains isolated no longer express this virulence factor [32, 33]. In order to determine whether this lack of Prn expression has an effect on moDC activation, six Prn deficient clinical isolates were included for moDC stimulation. No difference in the levels of all tested cytokines, including IL-10, G-CSF, TNF-α and IL-8 (Fig 6A–6D) were observed when comparing stimulation with strains that either express Prn or are deficient for Prn.

## Discussion

Despite high vaccination coverage, pertussis has reemerged affecting not only infants but also older individuals [35, 36]. Knowledge on how this bacterium persists in a vaccinated population is fundamental. Here, we aimed at determining the effect of emerging *B*. *pertussis* strains on innate immune responses. To this purpose, we investigated the activation of different human PRRs as well as moDCs by *B*. *pertussis* strains isolated from pertussis patients during the past 65 years in The Netherlands. HEK-Blue cells transfected with different PRRs were used as a high throughput screening system to detect activation of individual receptors by the *B*. *pertussis* strains. Findings indicate an increase in TLR2 activation by *B*. *pertussis* post-vaccination compared to pre-vaccination strains. TLR2 forms a heterodimer with either TLR1 or TLR6 and recognizes lipoproteins [37]. Recently, BP1569 and BP2992 from *B*. *pertussis* were identified as TLR2 activating lipoproteins [26]. A Blast search of 340 *B*. *pertussis* whole genome sequences [27] revealed no polymorphism for these genes in strains carrying the *ptxP1*, *ptxP2* or *ptxP3* alleles.These findings suggest that differential gene expression or post-translational modifications may explain the observed differences or that other TLR2 ligands are involved. For example, FHA has been proposed as a TLR2 ligand although this remains debatable [38, 39].

Modifications of the lipooligosaccharide (LOS) structure from *B*. *pertussis* strains has previously been reported to alter TLR4 signalling and DC activation [25, 40]. We observed no differences in TLR4 activation by strains from the three periods indicating no structural modifications of the LOS molecule from these *B*. *pertussis* strains. Other PRRs investigated in this study were NOD1 and NOD2. Murine NOD1 was previously shown to interact with tracheal cytotoxin (TCT), a *B*. *pertussis* toxin specifically destroying ciliated cells [41]. The authors reported that this virulence factor did not induce signaling of human NOD1, which is in line with our findings using live *B*. *pertussis*. Interestingly, we found that all the *B*. *pertussis* strains used in this study induced NOD2 signaling and that the post-vaccination strains induced significantly higher NOD2 activation compared to the pre-vaccination strains. To our knowledge, we are the first to show that live *B pertussis* can activate this PRR. NOD2 recognizes peptidoglycans containing the muramyl dipeptide moiety (*N*-acetylmuramyl-L-alanyl-D-isoglutamine) that are present in Gram-positive and negative bacteria [42]. Further studies are required to identify *B*. *pertussis* derived peptidoglycan moieties responsible for NOD2 activation. In addition, genomic, transcriptomic and/or proteomic studies may aid in determining whether the observed increased NOD2 activation is due to changes of gene expression or in the structure of the *B*. *pertussis* ligand(s) for NOD2.

Using the physiologically relevant moDCs, we observed differences in cytokine production as well as surface marker expression after stimulation with the different groups of *B*. *pertussis* strains. Findings indicate that post-vaccination strains induce increased DC activation. Interestingly, the most striking difference was an increase in the levels of the anti-inflammatory cytokine IL-10 produced by moDCs when stimulated with ACV-strains compared to Introd-Vac-strains. Besides its direct anti-inflammatory effect, this cytokine has been shown to be involved in the induction of regulatory T cells (Tregs) that play a role in controlling inflammation [43]. An increased anti-inflammatory response can benefit pathogen survival. For example, increased IL-10 production has been shown to be beneficial for *Bordetella parapertussis* (a Bordetella species also infecting humans) infection in mice by limiting a protective IFN-γresponse [44]. In addition to IL-10, we also observed an increase in G-CSF production by moDC upon stimulation with ACV-strains compared to Introd-Vac strains. G-CSF has also been implicated in the development of tolerogenic DCs and the induction of Tregs [45, 46]. Furthermore, upon comparison of several surface markers on moDC either stimulated with Introd-Vac- or ACV-strains, we detected an increased surface expression of the regulatory markers PD-L1 and CD101. The PD-L1 molecule on DCs can interact with PD-1 on T cells. PD1 is an inhibitory receptor, which is expressed on activated T cells and activation of this receptor results in regulation of T cell activation [47]. CD101 is known to inhibit T cell proliferation via the production of IL-10 by DCs [48]. The increased expression of these surface markers further strengthens our hypothesis that *B*. *pertussis* is evolving towards a phenotype that induces a stronger regulatory response.

In addition to the increased expression of regulatory markers, we observed increased expression of the activation markers CD80 and CD86 which is in accordance with the observed small but significant, increased production of IL-8 and TNF-α upon stimulation of moDC with the ACV-strains. The PRRs TLR2 and NOD2 have been reported to cross-talk and play a role in immune regulation as well as to influence the expression of one another [49]. TNF-α production has been shown to be enhanced upon simultaneous activation of NOD2 and TLR2, which could in part explain the increased TNF-α production by moDCs observed for ACV-strains compared to Introd-Vac strains [50]. Furthermore, cross-talk between TLR2 and NOD2 can induce anti-inflammatory signals in which IL-10 plays an important role [51, 52]. In order to address the role of TLR2 in the observed effect on moDCs by ACV-strains, we made use of a TLR2 blocking antibody. When blocking TLR2, although the levels of the cytokines were not altered, we observed that the expression of PD-L1 on these cells was reduced. This observation hints towards a role for TLR2 activation by *B*. *pertussis* in the expression of PD-L1 on these antigen presenting cells. This has previously been described for *Streptococcus pneumoniae*, that was shown to regulate PD-L1 expression on DCs in a TLR2 dependent manner [53]. Co-culture experiments using moDCs and T cells are required to determine whether these emerging strains indeed steer T cells towards a regulatory phenotype.

Over time P3 strains have emerged and make up over 90% of the currently circulating *B pertussis* population in Western countries [12]. To determine whether the observed DC phenotype induced by ACV-strains was associated with the emergence of the P3 allele, the strains used in this study were additionally divided into three groups namely, P1, P2 and P3. Although we observed an increased activation of TLR2 and NOD2 by P3 strains compared to P2 strains, no difference in cytokine production by moDCs, including IL-10, was detected. However, P3 strains isolated after the introduction of ACVs induced higher levels of IL-10 compared to P3 strains isolated before this time. This suggests that the increased anti-inflammatory cytokine induction by emerging *B*. *pertussis* strains cannot be attributed to carriage of the *ptxP3* allele. The presence of other polymorphic genetic loci, not necessarily linked to *ptxP3*, could be involved in the induction of IL-10. Whole genome sequencing of these strains may resolve this issue in future studies. Besides changes in *ptxP* alleles, changes in alleles encoding for Prn and fimbriae have also been reported [12]. Unfortunately, due to the low numbers of strains carrying similar sets of alleles, we were not able to determine whether combinations of alleles, or specific sequence types, were associated with the observed increased in anti-inflammatory cytokine production.

A most recent and striking adaptation of *B*. *pertussis* is the lack of expression of the vaccine antigen Prn. In countries were ACVs have been used for more than three decades, up to 85% of the circulating *B*. *pertussis* strains have been reported to no longer express Prn [30–34]. In 2015, the percentage of circulating Prn deficient strains was 14% in The Netherlands (Personal communication). This mutation has been suggested to be caused by (ACV) vaccine pressure, as Prn deficient strains show increased survival in mice vaccinated with ACVs compared to Prn-expressing strains [54, 55]. Furthermore, Prn-deficient strains are predominantly isolated from pertussis patients vaccinated with ACVs compared to unvaccinated patients [56, 57]. When we compared the cytokine profile of moDCs upon stimulation with strains either expressing or deficient in Prn, we found no differences. This finding indicates that the presence or absence of Prn in this pathogen has no effect on cytokine production by moDC.

The reemergence of pertussis has been attributed to several factors including bacterial strain adaptation [8]. Nonetheless, no studies have explored the effect of *B pertussis* strains emerging after the introduction of the pertussis vaccines on the host innate immune responses. This work pioneers in revealing a difference in innate immune activation by recently emerging strains compared to older strains on which our current vaccines are based. It also illustrates that the reporter HEK-blue cell lines expressing different PRRs can be used as screening platform to identify innate immune recognition and activation by *B*. *pertussis* strains.

Before the introduction of pertussis vaccination, *B*. *pertussis* mainly infected naïve individuals. Since nowadays most individuals have received a pertussis vaccination and thus have pre-existing vaccine-induced immunity to *B*. *pertussis*, this pathogen is in general no longer infecting a naïve host. Based on the findings here described, we propose that *B*. *pertussis* is evolving towards a phenotype that steer the host immune response towards a regulatory type, which could benefit bacterial survival and transmission in vaccinated hosts. This and further knowledge on innate immune activation by emerging strains is important to consider in the light of designing improved pertussis vaccines.

## Supporting Information

S1 Fig Control HEK-Blue Null (untransfected HEK-Blue cells) activation by B. pertussis strains and TLR ligands. HEK-Blue Null cells were stimulated with medium or live *B*. *pertussis* strains at a MOI of 40. The *B*. *pertussis* strains used were isolated either around the time the WCVs were introduced (Introd-vac-strains) or when the WCVs (WCV-strains) or ACVs (ACV-strains) were used. Furthermore, HEK-Blue Null cells were stimulated with one of the TLR ligands namely Pam3CSK4 (100ng/ml, TLR2), LPS-EK (100ng/ml, TLR4), iE-DAP (200ng/ml, NOD1) or MDP (200ng/ml, NOD2). HEK-Blue Null TLR activation was determined by measuring SEAP activity at an OD 650nm.
(TIF)

S2 Fig moDC activation by B. pertussis strains isolated from patients from 1949 to 2014. moDCs were stimulated with 29 different strains either isolated around the time the WCVs were introduced (Introd-Vac-strains) or when the WCVs (WCV-strains) or ACVs (ACV-strains) were used. MoDC activation measured by (A) IL-6, (B) MCP-1, (C) MIP-1-α and (D) MIP-1-β production by Introd-Vac-strains (1950–1960), WCV-strains (1970–2000) and ACV-strains (2005–2014). No IL-1β or IL-12 production could be detected. Medium stimulation values for IL-6, MCP-1, MIP-1-α, MIP-1-β and IL-1β are depicted in the corresponding graphs. To determine surface marker expression, moDCs were stimulated with 12 different *B*. *pertussis* strains isolated either around the time the WCVs were introduced (Introd-Vac-strains) or when the ACVs (ACV-strains) were used at a MOI of 10. MoDC surface expression of (E) CD97 and (F) CD172 by Introd-Vac-strains (1949–1960) and ACV-strains (2005–2014). Medium stimulation values for CD97 and CD172 are depicted in the corresponding graphs.
(TIF)

S3 Fig Activation of human TLR2, NOD2 and TLR4 by P3 WCV- and ACV- B. pertussis strains. HEK-Blue cell lines either expressing TLR2, TLR4, NOD2 or NOD1 were stimulated with P3 *B*. *pertussis* strains (MOI 40) isolated either when the WCVs (WCV-strains) or ACVs (ACV-strains) were used. TLR activation measured by SEAP activity of (A) HEK-Blue-TLR2 cells, (B) HEK-Blue-NOD2 cells or (C) HEK-Blue-TLR4 cells by P3-WCV-strains (1970–2000) and P3-ACV-strains (2005–2014). SEAP activity of cells in medium only or with the respective ligands, Pam3CSK4 (100ng/ml), LPS-EK (10ng/ml), MDP (200ng/ml) or iE-DAP (100ng/ml) are included for each HEK-Blue cell line.
(TIF)

S1 Table *B. pertussis* strain selection for moDC stimulation. Twenty-nine strains were selected as representative for each group based on their secreted embryonic alkaline phosphatase (SEAP) levels of PRR (TLR2, TLR4 and NOD2) activation represented as OD values. The average OD of the selected strains (Avg OD) did not deviate more than 15% from the average SEAP OD of all the strains in the respective group (Avg OD_allstrains_) (Introd-Vac, WCV or ACV-strains). Strains indicated in bold were used for the analysis of surface marker expression.
(PDF)
