# Supplementary figures and images for: Emerging Bordetella pertussis Strains Induce Enhanced Signaling of Human Pattern Recognition Receptors TLR2, NOD2 and Secretion of IL-10 by Dendritic Cells
Source: PLoS One. 2017 Jan 11;12(1):e0170027. doi: 10.1371/journal.pone.0170027 (PMC5226795; doi:10.1371/journal.pone.0170027)

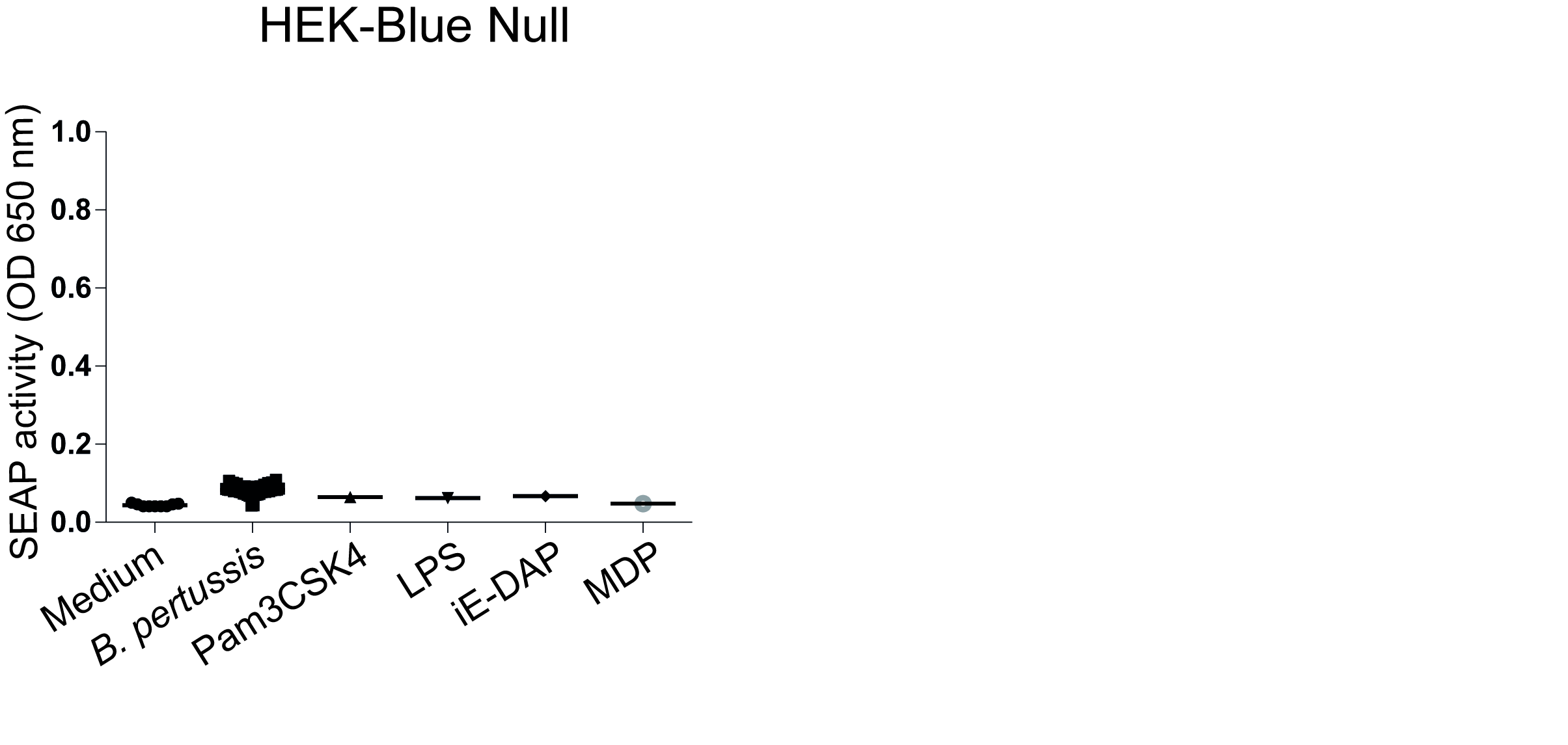

Supplement: S1 Fig — HEK-Blue Null cells were stimulated with medium or live B. pertussis strains at a MOI of 40. The B. pertussis strains used were isolated either around the time the WCVs were introduced (Introd-vac-strains) or when the WCVs (WCV-strains) or ACVs (ACV-strains) were used. Furthermore, HEK-Blue Null cells were stimulated with one of the TLR ligands namely Pam3CSK4 (100ng/ml, TLR2), LPS-EK (100ng/ml, TLR4), iE-DAP (200ng/ml, NOD1) or MDP (200ng/ml, NOD2). HEK-Blue Null TLR activation was determined by measuring SEAP activity at an OD 650nm. (TIF) [file pone.0170027.s001.tif]

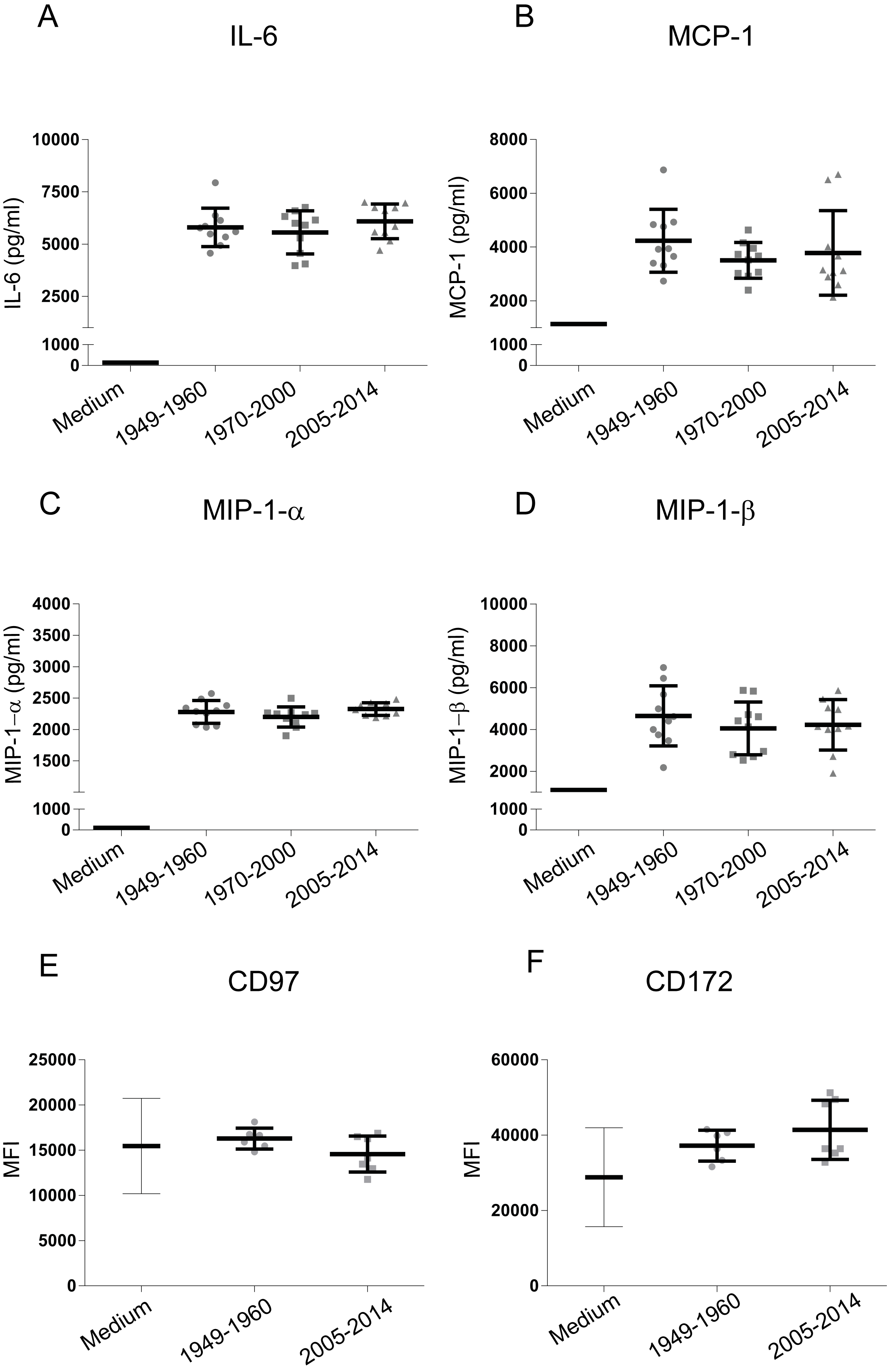

Supplement: S2 Fig — moDCs were stimulated with 29 different strains either isolated around the time the WCVs were introduced (Introd-Vac-strains) or when the WCVs (WCV-strains) or ACVs (ACV-strains) were used. MoDC activation measured by (A) IL-6, (B) MCP-1, (C) MIP-1-α and (D) MIP-1-β production by Introd-Vac-strains (1950–1960), WCV-strains (1970–2000) and ACV-strains (2005–2014). No IL-1β or IL-12 production could be detected. Medium stimulation values for IL-6, MCP-1, MIP-1-α, MIP-1-β and IL-1β are depicted in the corresponding graphs. To determine surface marker expression, moDCs were stimulated with 12 different B. pertussis strains isolated either around the time the WCVs were introduced (Introd-Vac-strains) or when the ACVs (ACV-strains) were used at a MOI of 10. MoDC surface expression of (E) CD97 and (F) CD172 by Introd-Vac-strains (1949–1960) and ACV-strains (2005–2014). Medium stimulation values for CD97 and CD172 are depicted in the corresponding graphs. (TIF) [file pone.0170027.s002.tif]

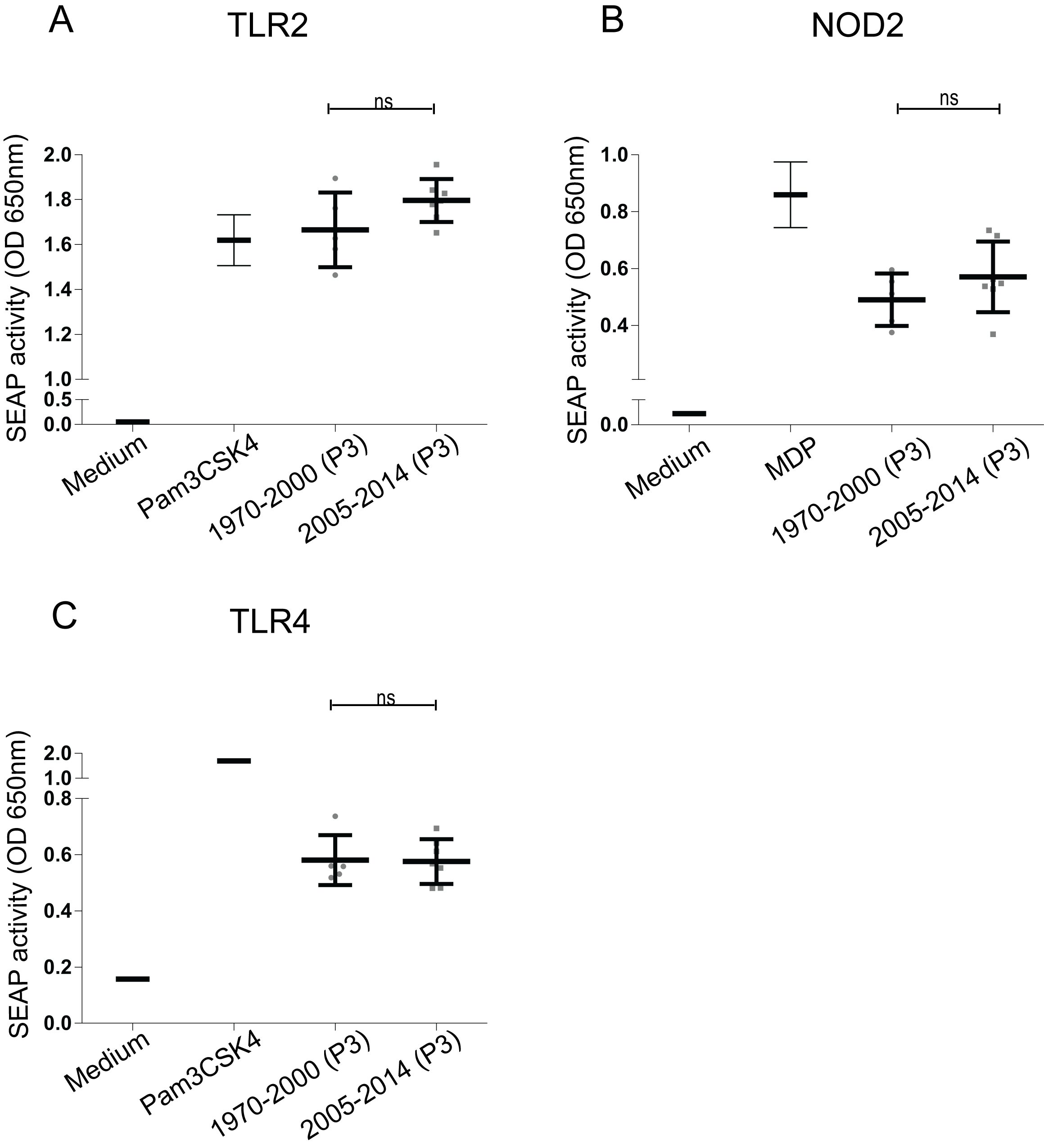

Supplement: S3 Fig — HEK-Blue cell lines either expressing TLR2, TLR4, NOD2 or NOD1 were stimulated with P3 B. pertussis strains (MOI 40) isolated either when the WCVs (WCV-strains) or ACVs (ACV-strains) were used. TLR activation measured by SEAP activity of (A) HEK-Blue-TLR2 cells, (B) HEK-Blue-NOD2 cells or (C) HEK-Blue-TLR4 cells by P3-WCV-strains (1970–2000) and P3-ACV-strains (2005–2014). SEAP activity of cells in medium only or with the respective ligands, Pam3CSK4 (100ng/ml), LPS-EK (10ng/ml), MDP (200ng/ml) or iE-DAP (100ng/ml) are included for each HEK-Blue cell line. (TIF) [file pone.0170027.s003.tif]
