# Supplementary material for: Emerging Bordetella pertussis Strains Induce Enhanced Signaling of Human Pattern Recognition Receptors TLR2, NOD2 and Secretion of IL-10 by Dendritic Cells
Source: PLoS One. 2017 Jan 11;12(1):e0170027. doi: 10.1371/journal.pone.0170027 (PMC5226795; doi:10.1371/journal.pone.0170027)
Supplement: S1 Table — Twenty-nine strains were selected as representative for each group based on their secreted embryonic alkaline phosphatase (SEAP) levels of PRR (TLR2, TLR4 and NOD2) activation represented as OD values. The average OD of the selected strains (Avg OD) did not deviate more than 15% from the average SEAP OD of all the strains in the respective group (Avg ODallstrains) (Introd-Vac, WCV or ACV-strains). Strains indicated in bold were used for the analysis of surface marker expression. (PDF) [file pone.0170027.s004.pdf]

Strain	Group	Isolation period (years)	SEAP activity (OD)		
			**TLR2**	**TLR4**	**NOD2**
B0558	Introd-Vac	1950-60	1.55	0.47	0.57
**B0304**		1950-60	1.75	0.36	0.52
**B0571**		1950-60	1.57	0.50	0.41
B1203		1950-60	1.85	0.62	0.55
**B1381**		1950-60	1.50	0.43	0.54
B1198		1950-60	1.34	0.49	0.56
**B0569**		1950-60	1.12	0.48	0.40
**B1378**		1950-60	1.37	0.43	0.54
**B1384**		1950-60	1.45	0.42	0.55
		**Avg (Avg $OD_{allstrains}$)**	**1.50 (1.51)**	**0.47 (0.51)**	**0.51 (0.51)**
B0440	WCV	1970-2000	1.70	0.56	0.44
B2939		1970-2000	1.58	0.47	0.51
B2965		1970-2000	1.54	0.59	0.46
B0354		1970-2000	1.94	0.54	0.53
B0599		1970-2000	1.68	0.47	0.63
B0541		1970-2000	1.63	0.52	0.60
B0606		1970-2000	1.76	0.56	0.42
B1960		1970-2000	1.89	0.74	0.38
B1895		1970-2000	1.58	0.53	0.55
B1917		1970-2000	1.46	0.56	0.51
		**Avg (Avg $OD_{allstrains}$)**	**1.67 (1.71)**	**0.55 (0.54)**	**0.50 (0.57)**
**B3212**	ACV	2005-nu	1.73	0.39	0.36
**B2914**		2005-nu	1.84	0.64	0.72
B3115		2005-nu	1.78	0.61	0.74
**B3135**		2005-nu	1.83	0.55	0.56
**B3446**		2005-nu	1.65	0.69	0.37
**B3896**		2005-nu	1.79	0.57	0.55
**B3962**		2005-nu	1.96	0.48	0.53
B4171		2005-nu	1.72	0.48	0.54
B3235		2005-nu	1.56	0.54	0.54
B3400		2005-nu	1.55	0.59	0.50
		**Avg (Avg $OD_{allstrains}$)**	**1.74 (1.71)**	**0.55(0.54)**	**0.54 (0.57)**
